# Project Stronger Together: Protocol to Test a Dyadic Intervention to Improve Engagement in HIV Care Among Sero-Discordant Male Couples in Three US Cities

**DOI:** 10.2196/resprot.7884

**Published:** 2017-08-31

**Authors:** Rob Stephenson, Nicolas A Suarez, Robert Garofalo, Marco A Hidalgo, Samuel Hoehnle, Jennie Thai, Matthew J Mimiaga, Emily Brown, Anna Bratcher, Taylor Wimbly, Patrick Sullivan

**Affiliations:** ^1^ Center for Sexual and Health Disparities University of Michigan Ann Arbor, MI United States; ^2^ Department of Health Behaviors and Biological Sciences School of Nursing University of Michigan Ann Arbor, MI United States; ^3^ Department of Health Behavior and Health Education School of Public Health University of Michigan Ann Arbor, MI United States; ^4^ Department of Pediatrics Feinberg School of Medicine Northwestern University Chicago, IL United States; ^5^ Ann & Robert H. Lurie Children’s Hospital of Chicago Chicago, IL United States; ^6^ Center for Health Equity Research Brown University Providence, RI United States; ^7^ Departments of Behavioral and Social Health Sciences and Epidemiology School of Public Health Brown University Providence, RI United States; ^8^ Department of Psychiatry and Human Behavior Alpert Medical School Brown University Providence, RI United States; ^9^ The Fenway Institute Fenway Health Boston, MA United States; ^10^ Department of Epidemiology Rollins School of Public Health Emory University Atlanta, GA United States

## Abstract

**Background:**

An estimated one- to-two-thirds of new human immunodeficiency virus (HIV) infections among US men who have sex with men (MSM) occur within the context of primary partnerships. Despite this fact, there remains a lack of prevention interventions that focus on male sero-discordant dyads. Interventions that provide male couples with skills to manage HIV risk, and to support each other towards active engagement in HIV prevention and care, are urgently needed.

**Objective:**

The objective of this paper is to describe the protocol for an innovative dyadic intervention (Stronger Together) that combines couples’ HIV testing and dyadic adherence counseling to improve treatment adherence and engagement in care among HIV sero-discordant male couples in the United States.

**Methods:**

The research activities involve a prospective randomized controlled trial (RCT) of approximately 165 venue- and clinic-recruited sero-discordant male couples (330 individuals: 165 HIV sero-negative and 165 HIV sero-positive). Couples randomized into the intervention arm receive couples’ HIV counseling and testing plus dyadic adherence counseling, while those randomized to the control arm receive individual HIV counseling and testing. The study takes place in three cities: Atlanta, GA (study site Emory University); Boston, MA (study site The Fenway Institute); and Chicago, IL (study site Ann & Robert H. Lurie Children’s Hospital of Chicago). Cohort recruitment began in 2015. Couples are followed prospectively for 24 months, with study assessments at baseline, 6, 12, 18, and 24 months.

**Results:**

Stronger Together was launched in August 2014. To date, 160 couples (97% of the target enrollment) have been enrolled and randomized. The average retention rate across the three sites is 95%. Relationship dissolution has been relatively low, with only 13 couples breaking up during the RCT. Of the 13 couples who have broken up, 10 of the 13 HIV-positive partners have been retained in the cohort; none of these HIV-positive partners have enrolled new partners into the RCT.

**Conclusions:**

The intervention offers a unique opportunity for sero-discordant couples to support each other towards common HIV management goals by facilitating their development of tailored prevention plans via couples-based HIV testing and counseling, as well as problem-solving skills in Partner Strategies to Enhance Problem-solving Skills (STEPS).

**Trial Registration:**

ClinicalTrials.gov NCT01772992; https://clinicaltrials.gov/ct2/show/NCT01772992 (Archived by WebCite at http://www.webcitation.org/6szFBVk1R)

## Introduction

Research has drawn attention to the role of male dyads in the US human immunodeficiency virus (HIV) epidemic, with primary partners identified as the source of approximately one-third [1] to two-thirds [2] of new HIV infections. Given these estimates, a significant paradigm shift in HIV prevention is needed, as efforts have traditionally focused on men who have sex with men (MSM), in particular gay-identifying men, as individuals rather than dyads. HIV prevention has predominantly emphasized HIV risks in the context of casual sex, largely ignoring the risk of HIV transmission that may occur within primary partnerships. Within male couples, various research findings have illustrated high rates of sexual risk behavior for HIV (with primary and casual partners), low rates of disclosure of potentially risky episodes with casual partners to primary partners, and reduced frequency of HIV testing [3-9]. Historically, HIV prevention efforts have focused on reducing the number of casual sex partners [10], indirectly messaging that primary partnerships pose reduced risk of HIV and conveying a misplaced sense of protection associated with primary partners [11,12]. However, there have been recent attempts to address this disproportionate focus on casual sex partners. The Office of the Global Acquired Immune Deficiency Syndrome (AIDS) Coordinator, through dissemination of prevention guidelines for MSM in the President’s Emergency Plan for AIDS Relief-supported countries, now recommends couples’ HIV testing and counseling (CHTC) for male couples [13].

CHTC has been used as an HIV prevention intervention for heterosexual couples in Africa for over 20 years [14]. Labeled as a, “ *high leverage HIV prevention intervention* ” by the US Centers for Disease Control and Prevention (CDC) [15], CHTC is considered to be an effective approach to HIV prevention among male couples. The difference between CHTC and individual HIV testing and counseling is that both partners receive counseling and testing at the same time, and do so together [16]. During the CHTC session, the counselor learns about the couple’s relationship and provides tailored counseling and HIV prevention recommendations based on the characteristics of the couple’s relationship and their joint HIV status [16]. Through the adaptation of CHTC and the high acceptability among MSM [14,17], preliminary data from MSM in three US cities (Atlanta, Chicago, Seattle) demonstrates the readiness of US MSM to receive and use CHTC [18,19]. Preliminary findings also suggest that male couples receiving CHTC, in which intimate partner violence (IPV) is not already present, do not report increased levels of IPV or relationship dissolution [20]. CHTC is now considered by the CDC to be an effective public health strategy, and is currently being implemented in over 40 US states [16,21].

A critical aspect of CHTC involves discussing a couple’s sexual agreement. Sexual agreements are common among male couples and refer to mutually understood rules between two partners that describe the kinds of sexual behavior that is allowed within and outside of the relationship [4,22-29]. In CHTC, male couples discuss their sexual agreements, role-play with the counselor about how they would communicate about a breach in the sexual agreement, and develop an HIV prevention plan based on their agreement and couple sero-status. Research regarding male couples’ agreements has demonstrated that men are less likely to practice condomless anal intercourse (CAI) with both primary and outside partners if they value and commit to their agreement and perceive their main partner to be dependable and invested in the relationship [28,30-32]. Promoting positive relationship dynamics has the potential to reduce couples’ risk for HIV because higher trust, communication, commitment, and social support are associated with lower odds of breaking a sexual agreement, which can ultimately reduce unique HIV risks (eg, CAI) for the couple [3].

In the United States, CHTC has been utilized as a prevention strategy for male couples to learn about their HIV sero-status together and to develop a tailored prevention plan that reflects both their sero-status and their sexual agreement. However, the potential for CHTC to be used as an entry point into engagement in HIV care has been largely overlooked. Increasing evidence indicates that early initiation of antiretroviral therapy (ART) is beneficial for both the HIV-positive person and his partner. Observational cohort studies have demonstrated a 94% decreased mortality risk for the HIV-positive partner with initiation of ART [33,34], as well as significant protection for the HIV-negative partner from HIV infection due to early ART initiation and progression to viral suppression [35]. Adherence to ART is critical, as resultant viral suppression is correlated with increased health [35-38] and reduced likelihood of HIV transmission to an HIV-negative person. Approximately 95% of ART adherence is the threshold required to achieve viral suppression, which is a threshold that is considerably higher than levels of medication adherence observed in many of the observational studies that examined the impact of viral suppression on HIV progression and transmission [39-41]. Only 41% of HIV-positive persons in the United States are both aware of their HIV infection and are undertaking ongoing HIV care [42]. Estimates suggest that only 77% of HIV-positive persons are linked to care and approximately half (51%) remain in care, with only 28% of HIV-positive persons in the United States achieving viral suppression [42]. Although a wide range of adherence rates (53-89%) have been documented in varied populations [40,43-52], the average rate of ART adherence is thought to be approximately 70% in the United States [42]. HIV-positive persons are also not receiving sufficient levels of HIV prevention counseling, and MSM are less likely than non-MSM to receive counseling in the preceding year [42].

There is evidence that dyadic interventions increase ART adherence when compared to individual adherence counseling [53]. In a randomized controlled trial (RCT) of 215 couples, including but not exclusively MSM couples, HIV-positive persons receiving ART adherence counseling with their partners had significantly higher levels of ART adherence [53]. The study examined the effects of joint counseling (in which counseling is delivered to the dyad) on adherence, but couples did not receive HIV testing together [53]. Social support among HIV-positive persons, including support from primary partners, is associated with fewer reported HIV risk behaviors with outside partners, greater self-efficacy to adhere, reported adherence, and lowered viral load after six months of follow-up [54,55]. Accordingly, couples who report higher relationship satisfaction are more likely to concur about the nature of their sexual agreement and to report not breaking the agreement [3,28].

In April 2012, World Health Organization released new guidelines for CHTC, including ART for treatment and prevention among sero-discordant couples [56]. The guidelines report a significant gap in evidence regarding the uptake and adherence to ART among sero-discordant couples, and highlight the role of CHTC in shaping uptake and adherence to ART [56]. This protocol outlines an intervention aimed at increasing engagement in HIV care among sero-discordant male couples in three US cities. The intervention draws upon two proven strategies to create a couples-focused package of care that incorporates dyadic HIV testing (CHTC) with dyadic adherence counseling. The intervention focuses on a couples-focused continuum of care, in which the couple is tested together and receives ART adherence counseling together, compared to a standard of care in which couples are tested and receive counseling individually. The RCT will examine and compare the intervention’s effects on engagement in HIV care and achievement of viral suppression for the HIV-positive member of the dyad, as well as sexual risk-taking both within and outside the dyad. Understanding the efficacy of a couple’s focused intervention for engagement in HIV care has the potential to inform the delivery of dyadic HIV prevention and care services for male couples, a group that is largely overlooked in current HIV prevention research and programming.

The intervention is grounded in Couple’s Interdependence Theory [57], a framework that combines both interdependence theory and communal coping perspectives. The framework guides the selection of measures of behaviors and behavior change within the couple. These measures relate to our intervention in two ways. First, some aspects of communication and decision-making within the partnership may influence the efficacy of the intervention; couples with more constructive communication styles may benefit more from CHTC and achieve greater linkage to, and retention in, HIV care than couples with less constructive communication styles. Second, some aspects of partnerships, such as efficacy around implementing behavioral change, may actually be modified by the intervention. In these cases, changes in key characteristics of the partnerships may be in the causal pathway between the intervention and the adoption of ART, linkage to care, and safer behaviors within the partnership. We thus conceptualize the causal pathways as follows. Couples exposed to the intervention package will receive opportunities to talk about HIV, safer sex, and care-seeking within their relationship jointly with a qualified CHTC counselor. Relative to couples exposed to individually-focused testing and adherence counseling, exposure to CHTC may in turn impact communal coping, use of coping, and transformation of motivation, leading to initiation and maintenance of health-enhancing behavior (which we conceptualize as greater uptake and retention in care and ART adherence), lowering of sexual risk-taking inside and outside of the relationship, and a resultant lowering of sexually transmitted infection (STI) and HIV incidence. In our research design, predisposing factors, outcome efficacy, and couple efficacy will be collected separately from both partners before the HIV testing intervention is delivered, and will again be collected at each of the follow-up visits.

## Methods

### Design

The research activities involve a prospective RCT of approximately 165 venue- and clinic-recruited sero-discordant male couples (330 individuals: 165 HIV sero-negative and 165 HIV sero-positive). The study takes place in three cities: Atlanta, GA (study site Emory University); Boston, MA (study site The Fenway Institute); and Chicago, IL (study site Ann & Robert H. Lurie Children’s Hospital of Chicago). Cohort recruitment began in 2015. Couples are followed prospectively for 24 months, with study assessments at baseline, 6, 12, 18, and 24 months.

### Participants

Eligible participants are cis-gender male couples in which: (1) two men report having been in a relationship with each other for greater than one month, with a relationship defined as, “having a male partner who you are committed to above all others”; (2) both men are aged over 18 years; (3) individuals have lived in the Atlanta, Boston, or Chicago metro areas for greater than 3 months; (4) participants reported no recent history (in the past 12 months) of IPV or coercion; and (5) an HIV sero-discordant relationship exists, in which both partners have disclosed their sero-status to each other. Prevalent HIV sero-positive statuses are self-reported and are not confirmed by study staff.

### Recruitment

Participants are recruited from the Atlanta, Boston, and Chicago metro areas via a multi-modal recruitment strategy. Recruitment takes place through physical and online/virtual spaces. Online sources include advertising on social media (eg, Facebook) and on geospatial dating apps (eg, Grindr). In-person recruitment is achieved by study staff attending lesbian, gay, bisexual, and transgender (LGBT) events, visiting venues, meeting potential participants at clinic appointments, and posting flyers in gay-themed venues. All recruitment activities provide individuals with the study uniform resource locator (URL). The online screener can also be administered by study staff in-person or over the phone. When men visit the URL, a page is populated containing a short description of study activities; if they express an interest in participation and provide the metro area they live in, potential participants are taken to the study consent form, and if they consent, are directed to a short eligibility screener. Men who (1) do not consent or (2) do not meet the eligibility criteria are taken to a screen thanking them for their interest. Eligible men are directed to a registration process. During the registration process individuals provide their name, email address, and a cell phone number. Participants are also given the option to provide their main partner’s email address and/or cell phone number so they can be contacted and screened to enroll the couple in the study together. Once both partners have (1) completed the consent forms, (2) finished the screening questionnaire, (3) been deemed eligible for the study, and (4) provided contact information, a staff member contacts the couple to schedule the couple for an in-person baseline visit.

### Check-In and Informed Consent

When an eligible couple comes in for a baseline visit, they are assigned an identification number and administered a “Check In” survey. This survey generates a randomization number, confirms eligibility, verifies the couple is a real couple, and gathers further contact information and alternative contacts for the participants. A real couple is considered two individuals who are devoted to each other above all others. This language is used in all questions that gather self-report of relationship status. If a couple is no longer eligible or determined not to be partnered, they are dismissed without study staff specifying why, in order to avoid instigating IPV or revealing eligibility criteria. Eligible couples are taken into separate rooms and read the consent forms by study staff who answer any questions the participants may have. If both participants in the couple give consent, the couple is enrolled and they begin the baseline survey. If one or both members of the couple decline consent, the couple is dismissed without study staff specifying eligibility criteria.

### Data Collection

After a couple gives informed consent, but before the couple is randomized to either the intervention or control group, each member of the couple is given a baseline survey. This survey is approximately 60-90 minutes long and collects data on demographics, relationship characteristics, sexual history, HIV care, and HIV prevention. In addition to survey data, biological samples are collected: STI testing, ART/preexposure prophylaxis (PrEP) drug adherence testing, and viral load testing. At the Boston site, however, STI testing is not conducted due to budgeting constraints.

### Randomization

Upon individual completion of the check-in survey by both partners, couples are randomized to either the control arm (Individual Counseling) or the intervention arm (Couples Counseling). The treatment assignments are generated with the use of one pseudo-random number generator across all three study sites. The randomization process generates a random number between 0 and 100. If given an odd number, the couple is placed in the control treatment group; if given an even number, the couple is placed in the intervention treatment group.

### Intervention

The intervention is a combination of CHTC and medication adherence counseling through the Partner Strategies to Enhance Problem-solving Skills (STEPS) method, which is an adaptation of an existing cognitive-behavioral intervention [16]. The intervention is comprised of three sessions. In the first session, lasting between 30-45 minutes, couples receive CHTC. The second and third sessions, lasting 60 minutes each, are held eight and ten weeks after the CHTC session, during which couples receive dyadic-focused ART adherence counseling. At the 6-, 12-, and 18-month follow-up visits, couples also receive Partner STEPS booster sessions.

#### Session One

CHTC sessions are conducted by a counselor who is trained in CHTC and last approximately 30-45 minutes. Only the HIV-negative partner is tested during the session. Posttest counseling focuses on dyadic prevention messages, and revisits the couple’s HIV risk concerns and sexual agreements in light of their test results. While focusing on the needs of the HIV-positive partner is necessary, the discussion also emphasizes how the couple can work together to keep the HIV-positive partner healthy and reduce transmission risks within the relationship. The prevention counseling element of the CHTC session focuses on talking to the couple about prevention options (including PrEP) and asking them to consider which prevention options may work best based on their relationship needs, context, and unique risk profile.

#### Sessions Two and Three

Couples in the intervention arm will attend two additional visits consisting solely of adherence counseling at 8 and 10 weeks after their first CHTC visit. Based on the efficacious Partner STEPS intervention, CHTC counselors utilize motivational interviewing to improve ART adherence among HIV-positive individuals. The Partner STEPS intervention was developed by drawing from relationship-oriented theory, existing efficacious individual-level ART adherence interventions, couple-focused HIV prevention interventions, and expert consultation. New content was incorporated to address all aspects of the HIV care continuum (eg, linkage to, and retention in, care) and to draw on relationship strengths through interactive activities. The theory-based Partner STEPS intervention is delivered by a trained bachelors-level counselor (interventionist). Each session is designed to use relationship strengths to increase motivation for HIV care and treatment, and cover sequential intervention *steps* relating to specific challenges in HIV care engagement and barriers to ART adherence. For each step, couples work with a trained interventionist to identify their unique challenges, actively problem-solve with the interventionist, and articulate and commit to working together to implement a plan in which each partner agrees to complete specific tasks. Partner STEPS counseling focuses on dyadic strategies to improve medication adherence and retention in care at each of ten *steps* for which Partner STEPS is named. Each step is a portion of HIV care that can present a challenge to those seeking care. The ten steps are: (1) transportation to appointments; (2) obtaining medications; (3) communicating with providers; (4) storing and transporting medications; (5) having a daily medication schedule; (6) coping with side effects; (7) adherence, self-care, and your relationship; (8) communicating within your relationship; (9) managing your social life and other relationships; and (10) dealing with privacy and disclosure. The counselors are trained to keep the focus on the couple by engaging both partners in problem solving and plan development. For sero-discordant couples, counselors are trained to focus the discussion on how *the couple* can *work together* to keep the HIV-positive partner healthy and to prevent transmission to the HIV-negative partner through medication adherence. Strategies to improve medication adherence and retention in care are tailored to the couple’s unique relationship, as the counselor asks the couple to consider strategies that may work best based on their relationship needs, context, and unique health situation. Bazzi et al describe the protocol for developing and testing of the Partner STEPS intervention [16].

### Control Group

Couples in the control group receive only one intervention visit, which is fewer than couples in the intervention. While it is possible that this aspect creates an attention effect, the control condition represents the current standard of care. At the baseline visit, the HIV-negative partner in the control group receives individual HIV counseling, testing, and referral (CTR). The HIV-positive partner receives information on the importance of ART uptake and adherence. Couples in the control arm do not receive Partner STEPS adherence counseling.

### Follow-Up Visits

All couples in the prospective cohort return for follow-up visits at 6, 12, 18 and 24 months following the baseline survey visit. These visits consist of a survey similar to the baseline survey, biological sample collection, and either CTR for the HIV-negative partner in the control arm, information on adherence to ART for the HIV-positive partner in the control arm (but not counseling on adherence), or CHTC and Partner STEPS counseling for the intervention couples. CHTC is offered to control arm couples at the 24-month visit. At the 24-month visit, a sample of 30 couples (10 per city) is also invited to participate in a brief qualitative exit interview that inquires into evaluation of the study experience and perceived effectiveness of the counseling they received. Over the course of enrollment, couples also receive bi-monthly phone calls to check-in on their relationship and assess medication adherence. These phone calls also serve to help with retention.

### Couple Dissolution

If a couple does not remain together throughout the follow-up period, HIV-positive partners will be retained in the study for the full 2-year follow-up period, while HIV-negative partners will return for one more follow-up visit and then will be censored from the cohort. If an HIV-positive partner in the intervention arm obtains a new partner who is eligible for the study, that partner will be invited to attend further follow-up visits, to continue providing CHTC to the original positive partner with the new partner. This new partner will participate in surveys, sample collections, counseling sessions and bi-monthly phone calls. At the Boston site, new negative partners are also invited to enroll if they are in the control arm.

### Biomedical Measures

For HIV-negative partners, HIV sero-status is tested at each study assessment. For HIV-positive partners, viral load and ART levels are measured at each study assessment and collected via dried blood spots. All participants are tested for syphilis at each study assessment in the Atlanta and Chicago sites. Budgetary constraints prevented testing for other STIs, as well as syphilis testing, in the Boston site.

### Outcomes

The primary outcomes are the HIV-positive partner’s engagement in HIV care and his achievement of viral suppression. Engagement in care is conceptualized as also including linkage to care and retention in HIV care. For HIV-positive partners who report no engagement in HIV care at baseline, linkage to care is defined as attending at least one clinical care appointment, having at least one CD4 test performed, and having at least one viral load test performed within 3 months of the baseline visit. Retention in care is measured by determining participation in continuous care; that is, at least two or more routine HIV visits at least three months apart, receiving two or more CD4 tests, and receiving two or more viral load tests within a 12-month period [58]. At each study visit, a blood draw will be conducted to provide a measure of the HIV-positive partner’s viral load and to test levels of ART. To supplement the viral load and ART-level biological markers, self-reported ART adherence is collected in each of the surveys. The AIDS Clinical Trial Group (ACTG) questionnaire includes items measuring adherence to medications for the past 4 days, adherence to scheduled instructions during the last weekend, and when any medication was last skipped. The questionnaire responses are weighted to calculate an adherence level from 0-100. The surveys also measure barriers to adherence via a 24-item scale based on the ACTG assessment for barriers to adherence to ART [59]. Participants are asked to note (using a scale ranging from *never* to *often*) if they missed their HIV medication over the past month for one of the provided reasons.

As secondary outcomes, the study measures sexual risk-taking and formation, and adherence of sexual agreements. For sexual agreements, participants are asked which of the following best describes their current sexual agreement with their main partner: (1) *both of us cannot have sex with outside partners*, (2) *we can have sex with outside partners, without any conditions or restrictions*, (3) *we can have sex with outside partners, but with some conditions or restrictions*, and (4) *we do not have an agreement*. Comparison of individual data will allow identification of discordant agreements. Participants will be asked if they have broken this agreement and whether this breakage was disclosed to their partner. To assess sexual behavior, measures adapted from the National HIV Behavioral Surveillance (NHBS) behavioral inventory collect information both on sexual behaviors with the main sex partner in the 3 months before the interview, and on sexual behaviors with all sex partners outside the relationship. For sex with the main partner, men are asked to estimate the number of anal sex acts with the main partner and the number of those acts that were condom-protected. For each partner outside the relationship, men are asked a series of questions to include characteristics of the outside partner, HIV status (if known), whether the sex outside the relationship was disclosed to the main partner, the number and type of sex acts, and the proportion of those sex acts that were protected by condoms. Additional outcomes assessed are centered on Couple’s Interdependence Theory.

#### Dyadic Characteristics

The four elements of Lewis’ model [57] (*predisposing factors of couples*, *partner’s transformation of motivation*, *process of communal coping*, and *use of communal coping*) are referred to as *dyadic characteristics*. In a recent RCT of CHTC, scales were developed to capture these constructs; all scales showed strong reliability, and evidence for construct validity was obtained for all scales [60]. In this intervention, each of the scales is collected in the baseline and follow-up surveys.

#### Predisposing Factors

Predisposing factors of couples uses several scales to measure this element. *Perceived Severity of HIV Scale:* this construct involves the perception of the personal, psychosocial, and physical consequences of a particular health threat. A total of 13 items were developed that crossed the three pertinent consequences of a particular health threat: personal, psychosocial, and physical. *Preferences for General Lifestyle Outcomes Scale*: this construct is defined as the degree to which interacting partners agree about the shared or joint outcomes in their relationship, which is composed of two subscales (*Preferences for General Lifestyle Scale* and *Preferences for Sexual Health Outcomes Scale*). The *Preferences for General Lifestyle Scale* includes six outcomes, including diet, nutrition, and social activities. *Preferences for Sexual Health Outcomes Scale* relates to sexual health (eg, reducing one’s risk for HIV). In addition, scales to measure other predisposing factors of couples are proposed for inclusion. *Conflict Style* determines how respondents typically handle conflict in their relationships, so the Conflict Style Inventory will be included [61]. *Communication Style* is measured with the Communication Patterns Questionnaire Constructive Communication subscale [62]. Finally, *Problem-Solving Skills* are measured with the adherence problem solving/readiness scale [63].

#### Partner’s Transformation of Motivation

In a recent RCT of CHTC, two measures were developed: ability of the participants to respond (1) cognitively and (2) emotionally to the health threat [20]. The scale for emotional response includes whether the respondent reports being fearful, nervous, or anxious about HIV. The scale for cognitive response includes whether the respondent reports understanding the risks of HIV transmission associated with being in a sero-discordant relationship, and the risks associated with outside sex partners.

#### Process and Use of Communal Coping

Several scales are used to measure this element. The *Outcome Efficacy to Reduce HIV Threat Scale* discusses how communal coping involves couples working together and making decisions to reduce the health threat. Three subscales were created to capture the full range of outcome efficacy related to these three processes of communal coping. For the first subscale, *Joint Effort*, the stem, “My partner and I believe that ‘working together’ versus on our own is an effective strategy” is used. For the second subscale, *Communication*, the stem, “Communicating with my partner is an effective strategy for” is used. For the third subscale, *Planning and Decision-making*, the stem, “My partner and I making decisions together rather than separately is an effective strategy” is used. The items for each of the three subscales were the same as the items used for the *Preferences for Sexual Health Outcomes Scale*. The *Couple Efficacy to Reduce HIV Threat Scale* defines couple efficacy as a couple’s confidence that together they can engage in communal coping efforts. The study also assesses the occurrence of violence within the relationship using the Conflict Tactics Scale Revised [64] to assess both perpetration and experience of IPV.

### Statistical Analyses

The analysis employs an intent-to-treat analysis design. The percentage of HIV-positive individuals who achieve viral suppression and report being fully engaged in care will be compared across arms, using Chi-square tests for significant difference. Retention in care is measured as the number of 6-month blocks during which at least one clinic visit was attended over the 2-year period following an initial attended visit; the percentage of HIV-positive individuals who report full retention in care (one clinic visit during each 6-month block) and the number of 6-month blocks during which care was received will be compared across study arms, using the appropriate tests for statistical significance. The capability of the intervention to yield longer-lasting effects in adherence endpoints over time will be examined. The visual analog scale data over the course of the study, at baseline and follow-up, will be analyzed using generalized linear models (GLMs) with properly-chosen (based on the distribution of dependent variables) link functions to analyze longitudinal adherence outcome data. The GLMs will be estimated using generalized estimating equations (GEE) with robust standard error estimates, which provide an extension of regression analyses to the cases of correlated or repeated observations and allows for inclusion of both categorical and count-dependent variables, and for appropriate modeling of covariance structures when observations are correlated across time. With appropriate link functions, GLMs can readily handle dependent variables with normal distributions, dichotomous outcomes, count data (Poisson distribution), and over-dispersed or zero-inflated count data (negative binomial models). The models will include the dyadic characteristics scales to examine the extent to which adherence is shaped by relationship functioning.

### Dyadic Characteristics: Analysis of the Scale Data Over Time

Repeated-measures analyses using mixed linear models will be performed for scale data. These analyses will include participant-level characteristics and couple-level variation or clustering. Participants will be nested within couples with the participant as the experimental unit. Repeated-measures analyses for each scale will be analyzed with a means model with SAS Proc Mixed providing separate estimates of the means by time on study and treatment group. An unstructured variance-covariance form among the repeated measurements will be assumed for each outcome, and estimates of the standard errors of parameters will be used to perform statistical tests and construct 95% confidence intervals. T-tests will be used to compare the pairwise differences between the model-based treatment means (least-squares means) at each time point. Statistical tests will be 2-sided. The model-based means are unbiased with unbalanced and missing data if missing data are noninformative (missing at random). A dropout process is assumed to be missing at random if, conditional on the observed data, the dropout is independent of the unobserved measurements.

### Incidence of Aggregate “Sex at Risk” Within Partnerships

At-risk sex will be defined as any CAI that occurs within the partnership during the follow-up period, even if the HIV-negative partner is taking PrEP or the HIV-positive partner has achieved viral suppression, in accordance with current CDC recommendations on PrEP and condom use [65]. The incidence of at-risk sex acts will be calculated as an incidence density, with the numerator being number of individual at-risk sex acts, and the denominator being person-years of follow time. Comparisons of the incidence of at-risk sex acts will be made by comparing incidence densities between the two arms. Incidence rates per couple-year of follow-up will be estimated and compared using exact methods based on the Poisson distribution when there are fewer than 15 events per subgroup or, when there are at least 15 events per group, by using the GEE approach. Baseline covariates include race, age, and duration of relationship. Period incidence rates (6-monthly incidence density rates) of at-risk sex will be estimated by performing a GEE Poisson regression analysis of the 6-monthly counts, implemented using the SAS PROC GENMOD procedure [66], and using an exchangeable correlation structure for the repeated observations of couples. The incidence density ratio (or incidence rate ratio; IRR) is the ratio of the incidence density in one treatment group (intervention arm) to that of a control group (standard of care). Results by each baseline covariate will be summarized as the IRR and the 95% confidence interval. In addition, we will tabulate data on disclosure of sex outside the relationship, the percentage of couples with agreements about sex outside the relationship, and the percentage of couples reporting agreement breakage or change in agreements. Prevalence of each outcome will be calculated, and prevalence of outcomes will be compared in the control and intervention groups using Chi-square tests or Fischer’s exact tests, as appropriate.

The analysis will also examine conceptual mediators and epidemiologically identified moderators. If the Stronger Together intervention works to increase viral suppression and number of participants reporting full engagement in care among the intervention sample in significantly greater magnitude than the comparison condition, we will assess the extent to which this relationship works through several possible mediators, including dyadic factors and relationship functioning (ie, communication). For mediation analyses, we will employ MEDIATE procedures. MEDIATE estimates the total, direct, and indirect effects of causal variable(s) on the outcome variable through a proposed mediator variable or set of mediator variables. For effect modification (moderation) analyses, we will add interaction terms one-by-one for the intervention condition and the potential moderators (eg, age, race/ethnicity, and psychosocial factors such as depression and length of relationship). Significant or large interaction terms would suggest that the effects of the intervention differ for different subgroups, as defined by the moderators.

The analysis will also examine HIV sero-conversion among HIV-negative partners and syphilis as secondary outcomes: the prevalence and 24-month cumulative incidence of HIV and syphilis will be examined in aggregate and then by study arm. During the interim assessment visits (that occur before month 12 and then again before month 24) we will collect information on STI testing/diagnosis/treatment that participants received elsewhere since their last study assessment visit. These data will be used to adjust analyses. Using Cox proportional hazard regression models, we will assess if intervention status results in decreased odds (hazard ratio) of HIV and STI infection, separately, over the 24-month period.

The safety of the intervention at the individual level will be assessed by examining reported IPV within the relationship and relationship dissolution. Prevalence of each individual adverse outcome or any adverse outcome will be calculated, and prevalence of outcomes will be compared in the control and intervention groups using Chi-square tests or Fischer’s exact tests, as appropriate.

### Incentives

Individual participants receive US $50 for completing each study visit; this includes baseline, Partner STEPS sessions, and all four-to-six follow-up visits, depending on the study arm. If both members of the couple complete all visits, the total incentive amount is US $500 per couple ($250 per individual participant) in the control and US $700 per couple ($250-350 per individual participant) in the intervention arm.

### Sample Size

Estimating 80% retention, we propose to enroll a sample of 165 male sero-discordant couples. The primary outcomes are engagement in HIV care and viral suppression. A sample of 165 sero-discordant couples provides statistical power (with 95% confidence and 80% power) to detect scientifically significant relative differences of 15%, 20%, and 25% in each of these outcomes between the two study arms. Additional health-enhancing behaviors include recent sexual risk-taking; using the data from our previous RCT of CHTC, this sample of 165 sero-discordant couples provides statistical power to identify significant differences in sexual risk-taking between the two arms. Using the methods described by Rosner for sample size estimation for longitudinal studies [67], a sample size of 75 couples in each group will ensure statistical power (using a two-sided two-sample t-test) to identify differences in the dyadic scale constructs between arms. As secondary outcomes, the study will examine sero-conversion and syphilis incidence between the two arms, although the power is insufficient to detect significance differences between the study arms.

### Trial Registration, Ethics, Consent, and Institutional Board Approval

The research and ethics presented in this study have been reviewed and approved by the Institutional Review Boards (IRBs) of Emory University (IRB #00065111), Lurie Children’s (IRB #2014-15896) and The Fenway Institute (IRB #FWA00000145), in addition to a Data Safety Monitoring Board that meets annually. The study is also registered on ClinicalTrials.gov (NCT01772992).

## Results

Stronger Together was launched in August 2014. To date, 160 couples (97% of the target enrollment) have been enrolled and randomized. The average retention rate across the surveys is 95%: current retention at 24 months is >90% across sites. Relationship dissolution has been relatively low, with only 13 couples breaking up during the RCT (13/160, 8%). Of the 13 couples who have broken up, 10 of the 13 (77%) HIV-positive partners have been retained in the cohort; none of these HIV-positive partners have enrolled new partners in the RCT.

## Discussion

Dyadic interventions provide an opportunity for male sero-discordant couples to learn the skills necessary to work together to manage HIV in their relationships. By developing tailored prevention plans in CHTC and developing problem solving skills in Partner STEPS, the intervention offers a unique opportunity for sero-discordant couples to support each other towards common HIV management goals. The intervention tested in this protocol builds off the current success of CHTC. The current intervention extends the focus of dyadic interventions for male couples across the continuum of HIV care, allowing male couples to develop the skills necessary to support active and successful engagement of HIV care. The intervention has the potential to fill a critical gap in efficacious interventions for male sero-discordant couples who, despite evidence of high rates of transmission within partnerships, have been largely ignored by HIV research and programming.

## Abbreviations

ACTG: AIDS Clinical Trial Group
AIDS: acquired immune deficiency syndrome
ART: antiretroviral therapy
CAI: condomless anal intercourse
CHTC: couples’ HIV testing and counseling
CTR: counseling, testing, and referral
GEE: generalized estimating equations
GLM: generalized linear model
HIV: human immunodeficiency virus
IPV: intimate partner violence
IRB: Institutional Review Board
IRR: incidence rate ratio
LGBT: lesbian, gay, bisexual, and transgender
MSM: men who have sex with men
NHBS: National HIV Behavioral Surveillance
PrEP: preexposure prophylaxis
RCT: randomized controlled trial
STEPS: Strategies to Enhance Problem-solving Skills
STI: sexually transmitted infection
URL: Uniform Resource Locator

## References

[1] Goodreau SM, Carnegie NB, Vittinghoff E, Lama JR, Sanchez J, Grinsztejn B, Koblin BA, Mayer KH, Buchbinder SP (2012). What drives the US and Peruvian HIV epidemics in men who have sex with men (MSM)?. PLoS One.

[2] Sullivan PS, Salazar L, Buchbinder S, Sanchez TH (2009). Estimating the proportion of HIV transmissions from main sex partners among men who have sex with men in five US cities. AIDS.

[3] Gomez AM, Beougher SC, Chakravarty D, Neilands TB, Mandic CG, Darbes LA, Hoff CC (2012). Relationship dynamics as predictors of broken agreements about outside sexual partners: implications for HIV prevention among gay couples. AIDS Behav.

[4] Hoff CC, Beougher SC, Chakravarty D, Darbes LA, Neilands TB (2010). Relationship characteristics and motivations behind agreements among gay male couples: differences by agreement type and couple serostatus. AIDS Care.

[5] Stephenson R, White D, Darbes L, Hoff C, Sullivan P (2015). HIV testing behaviors and perceptions of risk of HIV infection among MSM with main partners. AIDS Behav.

[6] Stall R, Mills TC, Williamson J, Hart T, Greenwood G, Paul J, Pollack L, Binson D, Osmond D, Catania JA (2003). Association of co-occurring psychosocial health problems and increased vulnerability to HIV/AIDS among urban men who have sex with men. Am J Public Health.

[7] Chakravarty D, Hoff CC, Neilands TB, Darbes LA (2012). Rates of testing for HIV in the presence of serodiscordant UAI among HIV-negative gay men in committed relationships. AIDS Behav.

[8] Mitchell JW, Petroll AE (2012). Patterns of HIV and sexually transmitted infection testing among men who have sex with men couples in the United States. Sex Transm Dis.

[9] Phillips G, Hightow-Weidman LB, Arya M, Fields SD, Halpern-Felsher B, Outlaw AY, Wohl AR, Hidalgo J (2012). HIV testing behaviors of a cohort of HIV-positive racial/ethnic minority YMSM. AIDS Behav.

[10] Kelly JA, St Lawrence JS, Diaz YE, Stevenson LY, Hauth AC, Brasfield TL, Kalichman SC, Smith JE, Andrew ME (1991). HIV risk behavior reduction following intervention with key opinion leaders of population: an experimental analysis. Am J Public Health.

[11] Pruitt KL, White D, Mitchell JW, Stephenson R (2015). Sexual agreements and intimate-partner violence among male couples. Int J Sex Health.

[12] Goldenberg T, Clarke D, Stephenson R (2013). “Working together to reach a goal”: MSM's perceptions of dyadic HIV care for same-sex male couples. J Acquir Immune Defic Syndr.

[13] (2011). The U.S. President's Emergency Plan for AIDS Relief (PEPFAR).

[14] Sullivan PS, Stephenson R, Grazter B, Wingood G, Diclemente R, Allen S, Hoff C, Salazar L, Scales L, Montgomery J, Schwartz A, Barnes J, Grabbe K (2014). Adaptation of the African couples HIV testing and counseling model for men who have sex with men in the United States: an application of the ADAPT-ITT framework. Springerplus.

[15] Painter TM (2001). Voluntary counseling and testing for couples: a high-leverage intervention for HIV/AIDS prevention in sub-Saharan Africa. Soc Sci Med.

[16] Bazzi AR, Fergus KB, Stephenson R, Finneran CA, Coffey-Esquivel J, Hidalgo MA, Hoehnle S, Sullivan PS, Garofalo R, Mimiaga MJ (2016). A dyadic behavioral intervention to optimize same sex male couples' engagement across the HIV care continuum: development of and protocol for an innovative couples-based approach (Partner Steps). JMIR Res Protoc.

[17] Wagenaar BH, Christiansen-Lindquist L, Khosropour C, Salazar LF, Benbow N, Prachand N, Sineath RC, Stephenson R, Sullivan PS (2012). Willingness of US men who have sex with men (MSM) to participate in Couples HIV Voluntary Counseling and Testing (CVCT). PLoS One.

[18] Stephenson R, Sullivan PS, Salazar LF, Gratzer B, Allen S, Seelbach E (2011). Attitudes towards couples-based HIV testing among MSM in three US cities. AIDS Behav.

[19] Neme S, Goldenberg T, Stekler JD, Sullivan PS, Stephenson R (2015). Attitudes towards couples HIV testing and counseling among Latino men who have sex with men in the Seattle area. AIDS Care.

[20] Sullivan PS, White D, Rosenberg ES, Barnes J, Jones J, Dasgupta S, O'Hara B, Scales L, Salazar LF, Wingood G, DiClemente R, Wall KM, Hoff C, Gratzer B, Allen S, Stephenson R (2014). Safety and acceptability of couples HIV testing and counseling for US men who have sex with men: a randomized prevention study. J Int Assoc Provid AIDS Care.

[21] Stephenson R, Grabbe KL, Sidibe T, McWilliams A, Sullivan PS (2016). Technical assistance needs for successful implementation of Couples HIV Testing and Counseling (CHTC) intervention for male couples at US HIV testing sites. AIDS Behav.

[22] Hoff CC, Beougher SC (2008). Sexual agreements among gay male couples. Arch Sex Behav.

[23] Crawford JM, Rodden P, Kippax S, Van de Ven P (2001). Negotiated safety and other agreements between men in relationships: risk practice redefined. Int J STD AIDS.

[24] Gass K, Hoff CC, Stephenson R, Sullivan PS (2012). Sexual agreements in the partnerships of Internet-using men who have sex with men. AIDS Care.

[25] Hosking W (2014). Australian gay men's satisfaction with sexual agreements: the roles of relationship quality, jealousy, and monogamy attitudes. Arch Sex Behav.

[26] Kippax S, Noble J, Prestage G, Crawford JM, Campbell D, Baxter D, Cooper D (1997). Sexual negotiation in the AIDS era: negotiated safety revisited. AIDS.

[27] Mitchell JW (2013). Characteristics and allowed behaviors of gay male couples' sexual agreements. J Sex Res.

[28] Mitchell JW, Harvey SM, Champeau D, Moskowitz DA, Seal DW (2012). Relationship factors associated with gay male couples' concordance on aspects of their sexual agreements: establishment, type, and adherence. AIDS Behav.

[29] Prestage G, Jin F, Zablotska I, Grulich A, Imrie J, Kaldor J, Honnor G, Kippax S (2008). Trends in agreements between regular partners among gay men in Sydney, Melbourne and Brisbane, Australia. AIDS Behav.

[30] Mitchell JW, Champeau D, Harvey SM (2012). Actor-partner effects of demographic and relationship factors associated with HIV risk within gay male couples. Arch Sex Behav.

[31] Mitchell JW, Garcia L, Champeau D, Harvey SM, Petroll AE (2012). HIV-negative seroconcordant gay male couples' attitudes, intentions, and perceived behavioral control for planned condom use within and outside of their relationships. Int J Sex Health.

[32] Mitchell JW, Harvey SM, Champeau D, Seal DW (2012). Relationship factors associated with HIV risk among a sample of gay male couples. AIDS Behav.

[33] Kitahata MM, Gange SJ, Abraham AG, Merriman B, Saag MS, Justice AC, Hogg RS, Deeks SG, Eron JJ, Brooks JT, Rourke SB, Gill MJ, Bosch RJ, Martin JN, Klein MB, Jacobson LP, Rodriguez B, Sterling TR, Kirk GD, Napravnik S, Rachlis AR, Calzavara LM, Horberg MA, Silverberg MJ, Gebo KA, Goedert JJ, Benson CA, Collier AC, Van Rompaey SE, Crane HM, McKaig RG, Lau B, Freeman AM, Moore RD, NA-ACCORD Investigators (2009). Effect of early versus deferred antiretroviral therapy for HIV on survival. N Engl J Med.

[34] Sterne JAC, May M, Costagliola D, de Wolf F, Phillips AN, Harris R, Funk MJ, Geskus RB, Gill J, Dabis F, Miró JM, Justice AC, Ledergerber B, Fätkenheuer G, Hogg RS, Monforte AD, Saag M, Smith C, Staszewski S, Egger M, Cole SR, When To Start Consortium (2009). Timing of initiation of antiretroviral therapy in AIDS-free HIV-1-infected patients: a collaborative analysis of 18 HIV cohort studies. Lancet.

[35] Cohen MS, Chen YQ, McCauley M, Gamble T, Hosseinipour MC, Kumarasamy N, Hakim JG, Kumwenda J, Grinsztejn B, Pilotto JHS, Godbole SV, Mehendale S, Chariyalertsak S, Santos BR, Mayer KH, Hoffman IF, Eshleman SH, Piwowar-Manning E, Wang L, Makhema J, Mills LA, de Bruyn G, Sanne I, Eron J, Gallant J, Havlir D, Swindells S, Ribaudo H, Elharrar V, Burns D, Taha TE, Nielsen-Saines K, Celentano D, Essex M, Fleming TR (2011). Prevention of HIV-1 infection with early antiretroviral therapy. N Engl J Med.

[36] Hogg RS, Heath KV, Yip B, Craib KJ, O'Shaughnessy MV, Schechter MT, Montaner JS (1998). Improved survival among HIV-infected individuals following initiation of antiretroviral therapy. JAMA.

[37] Palella FJ, Delaney KM, Moorman AC, Loveless MO, Fuhrer J, Satten GA, Aschman DJ, Holmberg SD (1998). Declining morbidity and mortality among patients with advanced human immunodeficiency virus infection. HIV Outpatient Study Investigators. N Engl J Med.

[38] Mocroft A, Ledergerber B, Katlama C, Kirk O, Reiss P, d'Arminio Monforte A, Knysz B, Dietrich M, Phillips AN, Lundgren JD, EuroSIDA study group (2003). Decline in the AIDS and death rates in the EuroSIDA study: an observational study. Lancet.

[39] Singh N, Berman SM, Swindells S, Justis JC, Mohr JA, Squier C, Wagener MM (1999). Adherence of human immunodeficiency virus-infected patients to antiretroviral therapy. Clin Infect Dis.

[40] Paterson DL, Swindells S, Mohr J, Brester M, Vergis EN, Squier C, Wagener MM, Singh N (2000). Adherence to protease inhibitor therapy and outcomes in patients with HIV infection. Ann Intern Med.

[41] Bartlett JA (2002). Addressing the challenges of adherence. J Acquir Immune Defic Syndr.

[42] Centers for Disease Control and Prevention (CDC) (2011). Vital signs: HIV prevention through care and treatment--United States. MMWR Morb Mortal Wkly Rep.

[43] Mannheimer SB, Matts J, Telzak E, Chesney M, Child C, Wu AW, Friedland G, The Terry Beirn Community Programs for Clinical Research on AIDS (2005). Quality of life in HIV-infected individuals receiving antiretroviral therapy is related to adherence. AIDS Care.

[44] Golin CE, Liu H, Hays RD, Miller LG, Beck CK, Ickovics J, Kaplan AH, Wenger NS (2002). A prospective study of predictors of adherence to combination antiretroviral medication. J Gen Intern Med.

[45] Bangsberg DR, Hecht FM, Charlebois ED, Zolopa AR, Holodniy M, Sheiner L, Bamberger JD, Chesney MA, Moss A (2000). Adherence to protease inhibitors, HIV-1 viral load, and development of drug resistance in an indigent population. AIDS.

[46] Wagner GJ, Kanouse DE, Koegel P, Sullivan G (2003). Adherence to HIV antiretrovirals among persons with serious mental illness. AIDS Patient Care STDs.

[47] Howard AA, Arnsten JH, Lo Y, Vlahov D, Rich JD, Schuman P, Stone VE, Smith DK, Schoenbaum EE, Her Study Group (2002). A prospective study of adherence and viral load in a large multi-center cohort of HIV-infected women. AIDS.

[48] McNabb J, Ross JW, Abriola K, Turley C, Nightingale CH, Nicolau DP (2001). Adherence to highly active antiretroviral therapy predicts virologic outcome at an inner-city human immunodeficiency virus clinic. Clin Infect Dis.

[49] Barroso PF, Schechter M, Gupta P, Bressan C, Bomfim A, Harrison LH (2003). Adherence to antiretroviral therapy and persistence of HIV RNA in semen. J Acquir Immune Defic Syndr.

[50] Ammassari A, Murri R, Pezzotti P, Trotta MP, Ravasio L, De Longis P, Lo Caputo S, Narciso P, Pauluzzi S, Carosi G, Nappa S, Piano P, Izzo CM, Lichtner M, Rezza G, Monforte A, Ippolito G, d'Arminio Moroni M, Wu AW, Antinori A, AdICONA Study Group (2001). Self-reported symptoms and medication side effects influence adherence to highly active antiretroviral therapy in persons with HIV infection. J Acquir Immune Defic Syndr.

[51] Ickovics JR, Meade CS (2002). Adherence to HAART among patients with HIV: breakthroughs and barriers. AIDS Care.

[52] Arnsten JH, Demas PA, Farzadegan H, Grant RW, Gourevitch MN, Chang CJ, Buono D, Eckholdt H, Howard AA, Schoenbaum EE (2001). Antiretroviral therapy adherence and viral suppression in HIV-infected drug users: comparison of self-report and electronic monitoring. Clin Infect Dis.

[53] Remien RH, Stirratt MJ, Dolezal C, Dognin JS, Wagner GJ, Carballo-Dieguez A, El-Bassel N, Jung TM (2005). Couple-focused support to improve HIV medication adherence: a randomized controlled trial. AIDS.

[54] Simoni JM, Frick PA, Huang B (2006). A longitudinal evaluation of a social support model of medication adherence among HIV-positive men and women on antiretroviral therapy. Health Psychol.

[55] Darbes LA, Chakravarty D, Beougher SC, Neilands TB, Hoff CC (2012). Partner-provided social support influences choice of risk reduction strategies in gay male couples. AIDS Behav.

[56] WHO (2012). Guidance on couples HIV counseling and testing including antriretroviral therapy for treatment and prevention in serodiscordant couples. WHO HIV/AIDS Programme.

[57] Lewis MA, McBride CM, Pollak KI, Puleo E, Butterfield RM, Emmons KM (2006). Understanding health behavior change among couples: an interdependence and communal coping approach. Soc Sci Med.

[58] Committee to Review Data Systems for Monitoring HIV Care (2012). Monitoring HIV care in the United States: IndicatorsData Systems. National Academies Press.

[59] Chesney MA, Ickovics JR, Chambers DB, Gifford AL, Neidig J, Zwickl B, Wu AW, Patient care committee & adherence working group of the outcomes committee of the Adult AIDS Clinical Trials Group (AACTG) (2000). Self-reported adherence to antiretroviral medications among participants in HIV clinical trials: the AACTG adherence instruments. AIDS Care.

[60] Salazar LF, Stephenson RB, Sullivan PS, Tarver R (2013). Development and validation of HIV-related dyadic measures for men who have sex with men. J Sex Res.

[61] Levinger G, Pietromonaco P (1989). Conflict Style Inventory. University of Massachusetts, Amherst.

[62] Heavey C, Larson B, Zumtobel D, Christensen A (1996). The communication patterns questionnaire: the reliability and validity of a constructive communication subscale. J Marriage Fam.

[63] Balfour L, Tasca GA, Kowal J, Corace K, Cooper CL, Angel JB, Garber G, Macpherson PA, Cameron DW (2007). Development and validation of the HIV Medication Readiness Scale. Assessment.

[64] Straus MA, Hamby SL, Boney-McCoy S, Sugarman DB (1996). The Revised Conflict Tactics Scales (CTS2). J Fam Issues.

[65] (2017). Centers for Disease Control and Prevention.

[66] Stokes M, Davis C, Koch G (2001). Categorical data analysis using the SAS system.

[67] Rosner B (2010). Fundamentals of Biostatistics.

